# Alarm tones, music and their elements: Analysis of reported waking sounds to counteract sleep inertia

**DOI:** 10.1371/journal.pone.0215788

**Published:** 2020-01-28

**Authors:** Stuart J. McFarlane, Jair E. Garcia, Darrin S. Verhagen, Adrian G. Dyer

**Affiliations:** 1 School of Media and Communication, RMIT University, Melbourne, Vic, Australia; 2 School of Design, RMIT University, Melbourne, Vic, Australia; IRCCS Istituto Delle Scienze Neurologiche di Bologna, ITALY

## Abstract

Sleep inertia is a potentially dangerous reduction in human alertness and occurs 0–4 hours after waking. The type of sound people set as their alarm for waking has been shown to reduce the effects of sleep inertia, however, the elemental musical factors that underpin these waking sounds and their relationships remain unclear. The goal of this research is to understand how a particular sound or music chosen to assist waking may counteract sleep inertia, and more specifically, what elements of these sounds may contribute to its reduction. Through an anonymous, self-report online questionnaire, fifty participants (*N = 50*) reported attributes of their preferred waking sound, their feeling towards the waking sound, and perceived sleep inertia after waking. This data enabled the analysis and comparison between these responses to identify statistically significant relationships. Our results did not return any significant association between sleep inertia and the reported waking sound type, nor the subject’s feeling towards their sound. However, the analysis did reveal that a sound which is ranked as melodic by participants shows a significant relationship to reports of reductions in perceived sleep inertia, and in contrast, sound rated as neutral (neither unmelodic nor melodic) returns a significant relationship to the reports of increases in perceived sleep inertia. Additionally, our secondary analysis revealed that a sound rated as melodic is considered to be more rhythmic than a melodically neutral interpretation. Together these findings raise questions regarding the impact melody and rhythm may hold with respect to sleep inertia intensity. Considering that the implementation of auditory assisted awakening is a common occurrence, the musical elements of a chosen waking sound may be an area to further interrogate with respect to counteracting sleep inertia.

## Introduction

*“The morning started disastrously*. *I slept through two alarms*, *one set for 0600 and another a half-hour later to remind me to take some CEO pictures*. *My body apparently went on strike for better working conditions*.”

**NASA astronaut journal report during orbit aboard the International Space Station**. **[1]**

Sleep inertia (*SI*) is a transitional sleep-wake phenomenon characterized by low arousal and reduced cognition [2, 3]. Initiated upon waking, *SI’s* symptoms can last for seconds, minutes or hours, where extended *SI* may impact human performance in a variety of situations and occupations [4–10]. This has been highlighted as a likely factor in the 2010 Air India Express air crash disaster that resulted in 158 fatalities. It has been shown that the captain of the aircraft had recently woken from an in-flight nap just prior to the crash. The poor decisions made after napping were attributed to the disaster, and have been linked to the effects of *SI* [6].

There has been growing research interest into the mechanics and architecture of *SI* [11–19], however the modalities and means to activate waking remains in its early stages with respect to this issue. YouGov [20] report that out of 586 participants surveyed, 68.2% use a form of alarm for waking, of these, 23% use an alarm clock, 14.9% a clock radio, and 26.3% an alarm on a cell phone. These figures show alarms are still an important means to assist in awakening, and given the 24-hour society in which we live and work, the need for peak performance from our waking device, the stimuli they produce, and the audio we select, is advantageous to counteract the negative effects of *SI*.

Research concerning human factors and psychology has provided initial insights into the application of countermeasures to reduce *SI*. Countermeasures are strategies or interventions to be implemented upon waking as opposed to methods that may consider pre-sleep hygiene techniques (routines to assist and promote sleep). Currently experimental countermeasures for *SI* include caffeine [21–25], light [24, 26–29], temperature [30, 31], post-awakening routines [24, 32], and sound [12, 33].

In the context of our research, two previous studies present findings on the effects of audio as a countermeasure for *SI*. Tassi et al [12] concluded that pink noise (75 dB) can reduce *SI* when deployed as an intense waking alarm [12], while Hayashi et al [33] discovered that excitatory music; particularly high-preference popular music (60 dB) as chosen by participants has the potential to reduce the intensity of *SI* after a short nap. Together, these authors provide evidence demonstrating that the use of sound and music may be beneficial in counteracting *SI*, however, a consensus as to the most appropriate type or design of stimuli to apply is undetermined.

Musical elements constitute terms and concepts recognized to assist in the description, understanding, and composition of sound and music [34–36]. In production, elements of music may be addressed individually or combined to produce a vast array of auditory aesthetics and compositions, including the types of songs we enjoy hearing, dance along with, and possibly awake to. Musical elements include, yet are not limited to; *pitch* (“The highness or lowness of a tone.”[37]); *volume* (“The degree of loudness.” [37, 38]); *tempo* (“The speed at which a passage of music is or should be played.” [37]); *rhythm* (“A strong, regular repeated pattern of movement or sound.” [37]); and *melody* (“A sequence of single notes that is musically satisfying; a tune.” [37]).

Pitch may be described as the perceptual correlate of the frequency at which a periodic tone completes one cycle and is measured in Hertz (Hz). Human frequency range spans approximately 20 Hz to 20000 Hz [39], while our ability to discern among deviations in pitch of a pure tone (a percentage with respect to the reference frequency) has been estimated to reside between a pitch variance of 2% and 4% [40].

Loudness (volume) is measured in decibels (dB), and in humans, the upper threshold of registration is approximately 140 dB [39, 41], with a discrimination range between 0.5 dB to 1.5 dB [40]. In environments where humans may typically sleep, dB may range between 40 dB (ambient conditions considered as quiet), 60 dB to 70 dB (as observed in office environments), 80 dB to 100 dB (car traffic noise), and approximately 110 dB if one resides near industrial building works [39, 41].

Psychological studies measure tempo in milliseconds (ms) while musical tempo is measured and notated in beats per minute (BPM). When defined by listeners, the preferred rate at which auditory stimuli proceed has been estimated to range between 500 ms (120 BPM) [42, 43] and 600 ms (100 BPM) [44]. Similarly, participants performing tapping sequences in a manner that they feel comfortable with (not too fast nor too slow) is accepted to have a range of approximately 300 ms (200 BPM) to 800 ms (75 BPM) [45–47] with a characteristic value of approximately 600 ms (100 BPM) [47]. Together, these results may be allied with the hypothesis of an innate internal timekeeper [48–54]. Lastly, humans are relatively adept at discriminating between tempo changes, which is enhanced with increased intervals at a faster tempo [55].

Rhythm may be defined as a sequence of discrete temporal intervals [47, 56, 57] where the rate of progression is often defined by a tempi. The ability to perceive rhythm has been hypothesized to be an innate human feature [58]. For example, infants display the capability to perceive variations in rhythm [59] and produce rhythmic movements [60]. Further, sleeping neonates can develop an expectation for the onset of rhythmic cycles [61].

Discrimination between rhythms has been shown to be performed with a high degree of accuracy in adults, and that perception of rhythm at slower rates differs from rhythm at faster rates [62]. Additionally, data suggests that listeners can distinguish between hypothetically unclear rhythms with acuity [63–65].

Perception of melody may formulate through assortments of pitch, tempo, and rhythm. Empirical studies show that melodies are easier to detect in expected patterns [66]; may be identified more readily if the pitches contained within the melody are sounded on the beat rather than off [67]; are memorized with greater accuracy when the tones with in a melody signify phrase endings [68]; and improve cognition if the contour (the rise and fall of tones within a melody) remains regardless of changes in octaves [69, 70]. Further, listeners may be able to detect melodic invariance when phrases are inverted, reversed (retrograded), and inverted and retrograded [71, 72]. Additionally, when listening to a sequence of pitches, participants can readily articulate expectations for how the melody will proceed [73–78]. It is hypothesized that the processing of melodic contour in memory may be universal among humans [79].

Musical elements are associated with the formulation of sound types and genres, though in comparison, afford the capacity to expose descriptive musical factors which may be neglected in sound type and genre classification [36, 80]. For example, and in the context of this research, a subject may describe their auditory awakening stimuli as ‘pop music’, however, when reported as musical elements, the audio may be defined as ‘very melodic’, ‘rhythmic’, ‘high pitch’, ‘fast tempo’, and ‘high volume’. From these elemental descriptions, ‘pop music’ may now be analyzed and understood in greater musical detail. In this way, the data may assist in the design of future sound stimuli that are further aligned with the listeners auditory experience.

This research explores waking audio and its relationship to *SI* through the deployment of a self-reporting online questionnaire designed to respond to three primary research questions; (i) ‘*Do waking sound types counteract the effects of SI*?*’*, (ii) ‘*Do subjective feelings towards waking sound types counteract the effects of SI*?*’*, and (iii) ‘*Do musical elements of waking sound counteract the effects of SI*?*’*.

To achieve our objectives, several separate analyses where performed comparing the respondents’ perceived *SI* against their waking sound types, subjective feelings, and the reported musical elements of their waking audio. Additional analyses include secondary level attributes of waking sound types and musical elements with respect to *SI*.

## Materials and methods

### Ethics statement

All research methods and data collection were approved by RMIT’s University College Human Ethics Advisory Network (CHEAN) (ref: CHEAN A 201710-02-17). The participants consisted of post-graduate students and staff members from the schools of Media and Communications, Industrial Design, and the School of Health and Biomedical Sciences, as well as the Australian Sleep Association. All respondents consented to participate by completing the online study. This was stipulated to the subjects in the ‘Invitation to participate’ email distributed during the recruitment period. Potential participants were encouraged to undertake the study without bias towards music aptitude or waking method. All respondents were 18 years of age and above consisting of males and females (See Results). In compliance with the study’s ethics application, and with respect to our specific research questions, age and gender have been recorded for demographics only and are not subject to analysis in this study. However, future investigations may consider pursuing gender and age comparisons.

### Data collection

Data was collected via a self-report online questionnaire. The submitted data was captured digitally via the use of the online software system Qualtrics [81], where the questionnaire was contained and operated. Qualtrics is specifically created for the undertaking of online questionnaires and surveys enabling researchers to design and implement their studies for ethically compliant distribution and data collection. The data obtained by Qualtrics is securely stored and only available for download and analysis by researchers with appropriate clearance.

### Questionnaire–Development and description

We designed a custom questionnaire which incorporated 14 items and 4 open ended response prompts. The questionnaire applied a combination of Likert scale, multiple choice and open-ended questions, to return qualitative and quantitative responses. The design comprises of four sections: Welcome Page (Section 1), Demographic Information (Section 2), Waking Sound (Section 3), and Sleep Inertia (Section 4), in total, requiring approximately ten minutes to complete. The ten-minute data collection was designed based on pilot results to minimize disruption to each subject during their natural ‘day-to-day’ waking routine, so as to maximise the ecological validity of the result. Please refer to the supporting material (S1 Table) for a transcript of the entire questionnaire.

Section 1 is an introduction to the study and clarifies the participants’ obligations prior to completing the task. This includes a reminder that by completing and submitting the survey the participant has given their specific consent to partake in the study.

Section 2 (Items 1–5) is comprised of multiple-choice (Items 1–3) and Likert scale (bipolar Item 4, unipolar Item 5) questions, which gather the demographic data of the respondents, their music appreciation, and musical aptitude. This data is paired with responses from Section 3 (Items 7–9, 11 (Volume) - 12) and reported in the results.

Section 3 gathers information relating to the respondents use of audio for waking (if applicable), and the reported musical detail of the audio. This section incorporates Likert scale, multiple choice and open-ended questions. The first item in Section 3 (Item 6, multiple choice) identifies whether respondents use audio to assist them in waking. If the participant does not, they are prompted to describe their waking method, then forwarded to Item 14 in Section 4. If the participant does use audio, they are required to report what device is preferred to play the audio (Item 7, multiple choice), the number of days per week at which audio is employed (Item 8, multiple choice), and consistency of use (Item 8, multiple choice). When compiled, these items contribute to the participant profile (Alarm adoption & application) previously outlined in the description of Section 2.

Item 10, ‘Which most frequently used audio for waking up best represents yours?’ requires the participants to nominate or specify the type of audio they use from six options provided. This item is the essential component to determine each participants’ waking sound type, and is elemental in formulating the analysis in response to primary research question (i) *‘Do waking sound types counteract the effects of SI*?*’*.

In designing the options for item 10, we first defined ‘sound type’ [82, 83] as the most suitable overarching terminology in this context to define all audio that may be reported as waking sound stimuli (e.g. musical genre, auditory tone, white or pink noise, human speaking, the sound of the wind, or aircraft engine noise). Secondly, the first five options for selection (Alarm tone, Musical song, Instrumental music, Natural sounds, Radio) were elected as they are familiar descriptions of audio sound types that respondents may use for waking. To determine the categories, we surveyed available pre-set sound types and custom audio functionality provided by several device manufacturers [84–90]. The sixth option (Other) allows for the respondent to describe their specific sound type if desired. In sum, these options allow for the breadth of potential waking sound types respondents may report.

Operationally, if ‘Musical song’ or ‘Instrumental music’ are selected, the respondent is then forwarded to Item 10.1 and are requested to specify which genre represents their waking sound type. The categories of genre for selection have been adapted from the Short Test of Music Preference (STOMP) [91]. Similarly, when ‘Radio’ is specified the respondent is prompted for the specific station. If ‘Other’ is selected, the participant is requested for a description.

Item 11 is another key factor of the study and is employed to analyse primary research question (iii) ‘*Do musical elements of waking sound counteract the effects of SI*?*’*.

This item gathers each respondent’s classification of their waking audio’s musical elements which is used to establish a profile of the stimuli from a fundamental musical level. Each participant is required to rank the musical elements of their waking audio on a 5-point bipolar Likert scale that we developed. These specific ranks have been selected to afford descriptions of the participant’s waking audio musical elements through subjective interpretations (e.g. Negative, Neutral, Positive). Ranks 1, 3, and 5 are labelled (i.e. 1 = Unmelodic, 3 = Neither unmelodic nor melodic, 5 = Very melodic), while rank 2 and 4 remain uncategorized to reduce respondent bias. When reporting and discussing the results, we included labels to ranks 2 and 4 for continuity (i.e. rank 2 = Somewhat unmelodic, Somewhat nonrhythmic, Somewhat slow in tempo, Somewhat low in pitch, Somewhat low in volume, and rank 4 = Melodic, Rhythmic, Fast tempo, High pitch, High volume).

Section 3 is completed with Items 12 and 13. Item 12 (multiple choice) requests if the respondent’s waking audio rises in volume over time (a detail relating to contemporary sound design and increasingly common as an option available in many of today’s alarm devices and applications [84–88]). This item’s results are contained in the ‘Alarm adoption & application’ demography.

Item 13 investigates the respondents’ subjective feelings towards their waking sound and allows for the statistical analysis of primary research question (ii) ‘*Do subjective feelings towards waking sound types counteract the effects of SI*?*’*.

Deployed as a 5-point bipolar Likert scale, this design rates the participant’s subjective feeling towards their waking sound as: Very pleasant, Pleasant, Neutral, Unpleasant, and Very unpleasant.

Section 4 is the leading component to the ‘Waking Sound and Sleep Inertia’ questionnaire as it enables the statistical reporting of respondents’ *SI* intensity. Adapted from the Sleep Inertia Questionnaire (SIQ) developed by Kanady and Harvey [92], the SIQ has been researched and analysed to be a reliable measure of *SI* [92, 93]. Our adapted SIQ begins with the question; ‘After you wake up, to what extent do you…’ [92] and is followed by each item.

All respondents are required to rate each item as either, Not at all = 1, A little = 2, Somewhat = 3, Often = 4, All the time = 5. In the context of this study, eight items were removed from the original SIQ questionnaire as they are already included in other items in the questionnaire, or are not a focus of this study.

### Procedure

The project was enabled through an anonymous online questionnaire that is performed after waking by each participant. This method was chosen to maximise the natural contextual environment in which subjects use auditory alarms. Subjects were invited to participate via email through their respective schools or member association. This method for distribution was defined to ensure the anonymity of the participants. The contents of the email included an introduction, title and overview of the research, who is conducting the study, participants’ rights and responsibilities (including a requirement of normal hearing), instructions for how to undertake the questionnaire, and contact information for any further enquiries regarding the test. It is stipulated that the subjects are required to complete the questionnaire within four hours after waking, and that all questions must be responded to when prompted. Furthermore, the invitation email explicitly states that by completing the questionnaire, the participants are agreeing to undertake the research. By so doing, the invitation email effectively replaces a traditional participant information ‘hard copy’ form and serves as the online equivalent. The invitation to participate included a link to the study which directed each respondent to the online questionnaire for commencement. The study was launched during May 2017 and concluded in May 2018.

### Data processing

The total number of initial respondents was 83 (*N* = 83) which was filtered omitting any inconsistent or incomplete responses, reducing *N* to (*N* = 73). Further, this study requires the analysis of waking sound stimuli, therefore the ‘No waking sound’ responses were disregarded, reducing *N* to (*N* = 50). The measure of perceived *SI* intensity is determined as the mode of each participant’s responses to all of the SIQ questionnaire items in Section 4. The mode was selected as a measure of central tendency as it is consistent with the SIQ categories (i.e. Not at all = 1, A little = 2, Somewhat = 3, Often = 4, All the time = 5), is an example of ordinal data, and hence, the statistical adjustments required to calculate the mean and standard deviation are inappropriate for ordinal data [94, 95]. The raw data was initially filtered through Microsoft Excel [96], then imported to SPSS [97] for statistical analysis.

### Statistical analysis

#### Primary analysis

To address the three primary research questions, (i) ‘*Do waking sound types counteract the effects of SI*?*’*, (ii) ‘*Do subjective feelings towards waking sound types counteract the effects of SI*?*’*, and (iii) ‘*Do musical elements of waking sound counteract the effects of SI*?*’*, we conducted a series of test analyses. The results from the adapted SIQ (Item 14) are analysed against the data sets obtained from Items 10, 13, and 11. Table 1 contains the sequence of analyses, and each test we performed for the primary research questions.

**Table 1:** Primary test analysis.

Test analysis	Primary research question	Analysis comparisons
**No 1.**	(i) ‘Do waking sound types counteract the effects of *SI*?’	*SI* (Item 14) vs Sound type (Item 10)
**No 2.**	(ii) ‘Do subjective feelings towards waking sound types counteract the effects of *SI*?’	*SI* (Item 14) vs Subjective feeling (Item 13)
No 3.	(iii) ‘Do musical elements of waking sound counteract the effects of *SI*?’	*SI* (Item 14) vs Music element—Melody (Item 11)
No 4.	(iii) ‘Do musical elements of waking sound counteract the effects of *SI*?’	*SI* (Item 14) vs Music element—Rhythm (Item 11)
**No 5.**	(iii) ‘Do musical elements of waking sound counteract the effects of *SI*?’	*SI* (Item 14) vs Music element—Tempo (Item 11)
**No 6.**	(iii) ‘Do musical elements of waking sound counteract the effects of *SI*?’	*SI* (Item 14) vs Music element—Pitch (Item 11)
No 7.	(iii) ‘Do musical elements of waking sound counteract the effects of *SI*?’	*SI* (Item 14) vs Music element—Volume (Item 11)

Each primary test analysis was first trialled with a contingency table analysis (Pearson’s Chi Square Test) [98] to determine significant relationships between the measure of perceived *SI* and each factor. For analyses with significant relationships and a cell count less than five, Fisher’s Exact Test [99] was then performed utilising the Exact Monte Carlo method using 100000 repeated random samples [100]. Adjusted cell residuals were generated for the significant analyses. Residuals where converted into z-scores related to the probability of observing the respective value under the null hypothesis of independence.

#### Secondary analysis

For the significant (*p* < 0.05) results gathered from the primary analysis, we performed a secondary series of analyses. On this condition, we tested the appropriate items (10, 11, 13) against each significant result obtained. By conducting this sequence of analyses, we can respond to the primary research questions by defining secondary level interactions between the primary results and each conditionally relevant item (i.e. sound type Item 10, subjective feeling Item 13, and music elements Item 11). These results provide data to be considered in the formulation and design of waking audio for *SI* in future studies. Each secondary analysis performed can be viewed in Table 2.

**Table 2:** Secondary analysis: Melody vs sound type and musical elements.

Test analysis	Analysis comparisons
**No 8.**	Music element—Melody (Item 11) vs Sound type (Item 10)
**No 9.**	Music element—Melody (Item 11) vs Music element—Rhythm (Item 11)
**No 10.**	Music element—Melody (Item 11) vs Music element—Tempo (Item 11)
**No 11.**	Music element—Melody (Item 11) vs Music element—Pitch (Item 11)
**No 12.**	Music element—Melody (Item 11) vs Music element—Volume (Item 11)

Each secondary analysis (Table 2) was first tested with a contingency table analysis (Pearson’s Chi Square Test) to determine significance against the conditional factors. Results of significance progressed for further analysis, while nonsignificant results were omitted. See Table 3.

**Table 3:** Secondary analysis cases of significance.

Test analysis	Analysis comparisons
**No 8.**	Music element–Melody (Item 11) vs Sound type (Item 10)
**No 9.**	Music element—Melody (Item 11) vs Music element–Rhythm (Item 11)

For each test analysis in Table 3 we implemented the Fisher’s Exact Test utilising the Exact Monte Carlo method using 100000 repeated random samples. Adjusted cell residuals were generated for the significant Fisher’s Exact Test analyses. Residuals where converted into z-scores related to the probability of observing the respective value under the null hypothesis of independence.

## Results

### Demographics

Tables 4, 5 & 6 show the counts and percentages of the raw data for the ‘Respondents’, ‘Music appreciation & aptitude’, and **‘**Alarm adoption & application’ demographic categories.

**Table 4:** Respondents.

Item	Category	Count	%
**Age group**	18–29	14	28
	30–39	12	24
	40–49	11	22
	50–59	10	20
	60+	3	6
**Gender**	Male	15	30
	Female	35	70

**Table 5:** Music appreciation & aptitude.

Item	Category	Count	%
**Average music listening per day**	0–30 minutes	19	38
	31 minutes—1 hours	17	34
	1–3 hours	9	18
	3+ hours	5	10
**Music appreciation**	Like a great deal	31	62
	Like somewhat	17	34
	Neither like nor dislike	2	4
	Dislike somewhat	0	0
	Dislike a great deal	0	0
**Accuracy in describing music**	Extremely well	7	14
	Very well	18	36
	Moderately well	17	34
	Slightly well	7	14
	Not well at all	1	2

**Table 6:** Alarm adoption & application.

Item	Category	Count	%
**Device to play the audio**	Clock Radio (alarm)	7	14
	Mobile phone	42	84
	Tablet	1	2
	Laptop	0	0
	Other	0	0
**Days per week audio is used to wake up**	1.	3	6
	2.	2	4
	3.	1	2
	4.	4	8
	5.	17	34
	6.	11	22
	7.	12	24
**Same audio to wake up every day**	Yes	46	92
	No	4	8
**Volume increase over time**	Yes	17	34
	No	33	66
**Reported volume level**	Low volume	2	4
	Somewhat low in volume	9	18
	Neither low volume nor high volume	22	44
	High volume	12	24
	Very high volume	5	10

### Primary analysis

#### Test analysis No 1: *SI* vs Sound type

**Fig 1:**
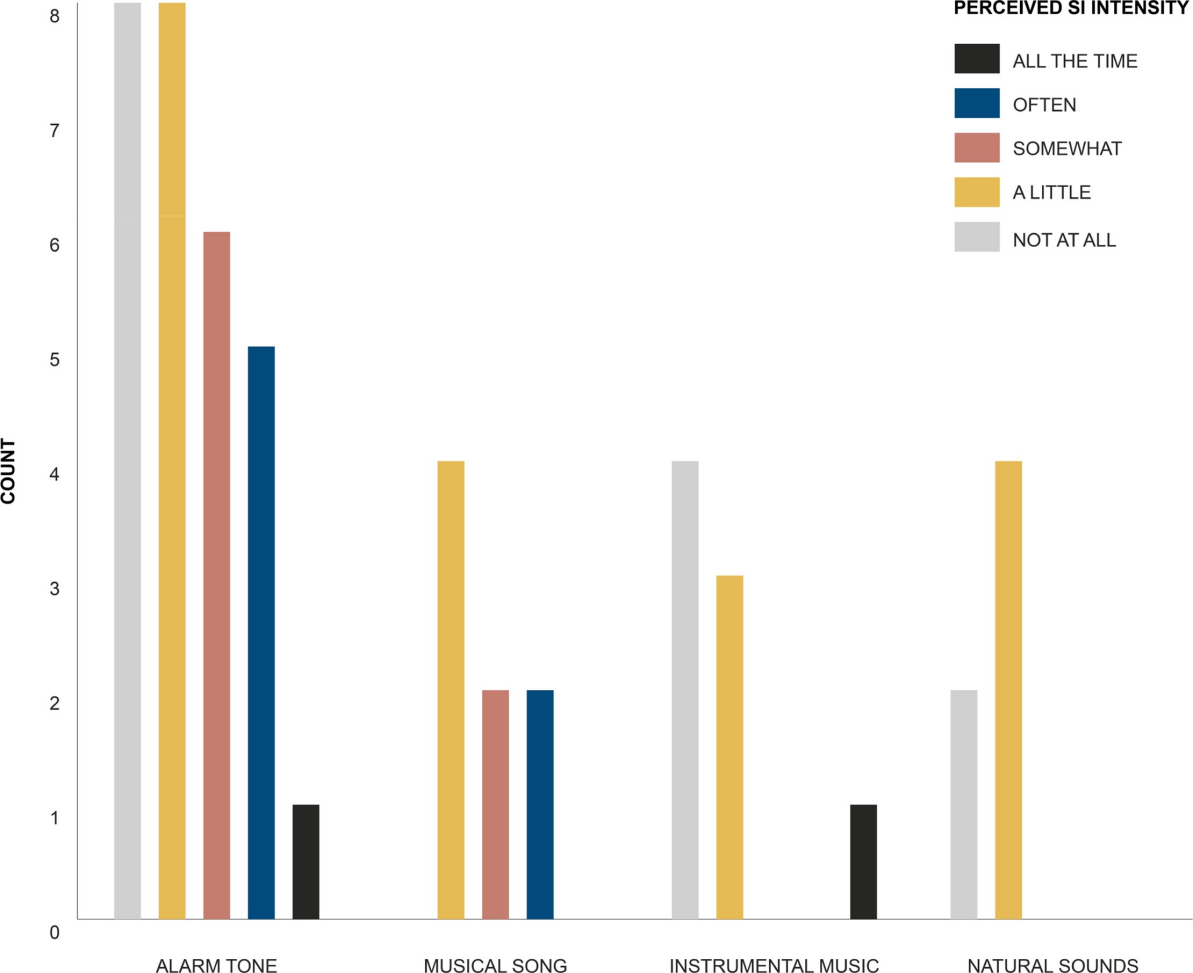
Counts of perceived SI intensity against participants reported waking sound type.

In our sample of *N* = 50 individuals the contingency table analysis (Pearson’s Chi Square Test) showed that there was no significant relationship between the measure of perceived *SI* intensity and the participants waking sound type *X*^*2*^ (12, *N* = 50) = 14.07, *p* = 0.296. See Fig 1 for the counts of perceived *SI* intensity against participants reported waking sound type.

#### Test analysis No 2: *SI* vs Subjective feeling

**Fig 2:**
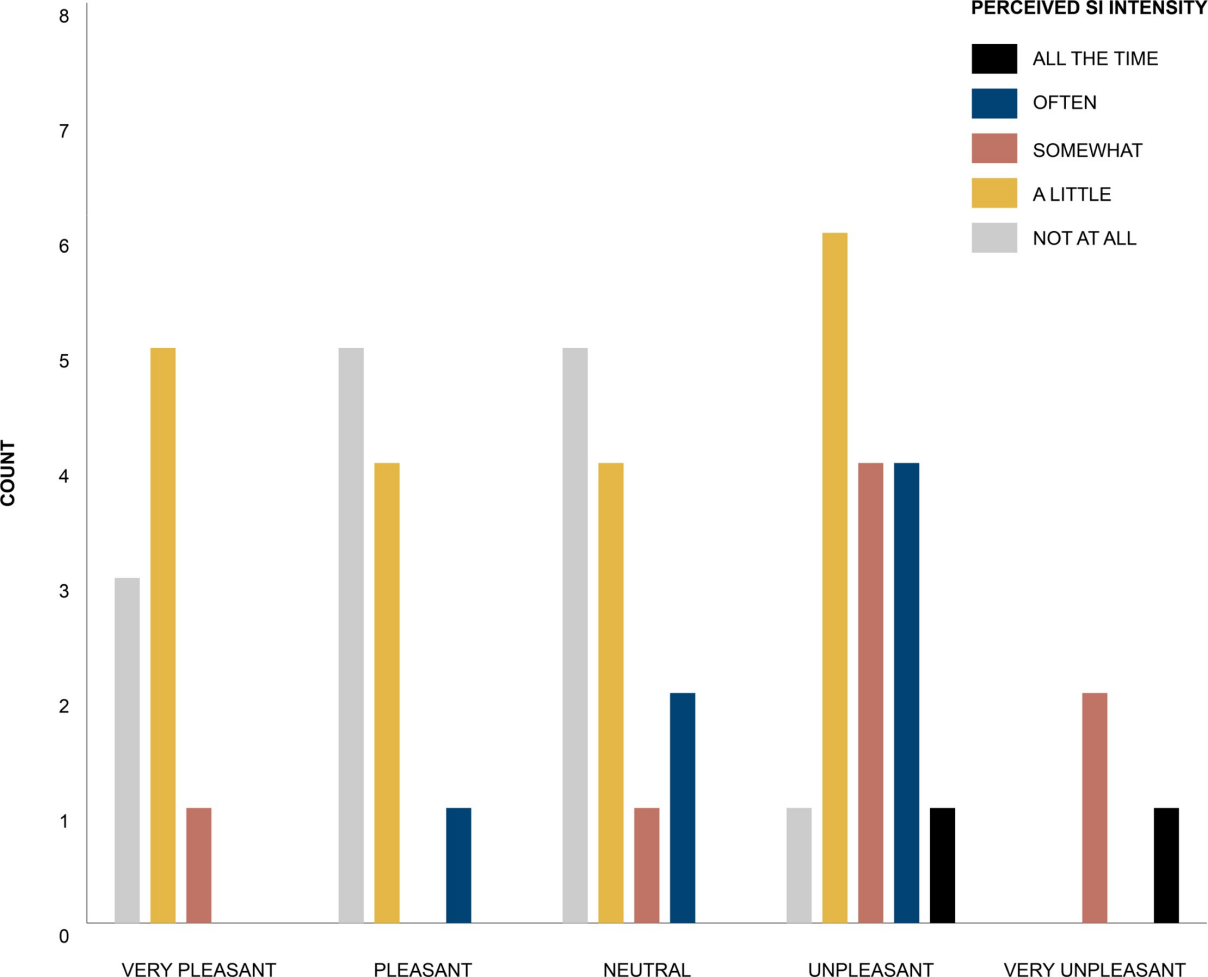
Counts of perceived SI intensity against participants reported feeling towards their waking sound type.

Fisher’s Exact Test showed that there was no significant relationship between the measure of perceived *SI* intensity and the participants subjective feeling towards the waking sound *X*^*2*^ (*N* = 50) = 21.05, *p* = 0.068. See Fig 2 for the counts of perceived *SI* intensity against participants’ reported feeling towards their waking sound type.

#### Test analysis No 3: *SI* vs Musical element (Melody)

The initial contingency table analysis (Pearson’s Chi Square Test) showed that there was a significant relationship between the measure of perceived *SI* intensity and the reported melodicity of the subject’s waking sound *X*^*2*^ (16, *N* = 50) = 26.77, *p* = 0.044. This result was also confirmed by the Fisher’s Exact Test *X*^*2*^ (*N* = 50) = 23.54, *p* = 0.023 with V = 0.37 corresponding to a moderately strong effect size [101].

**Fig 3:**
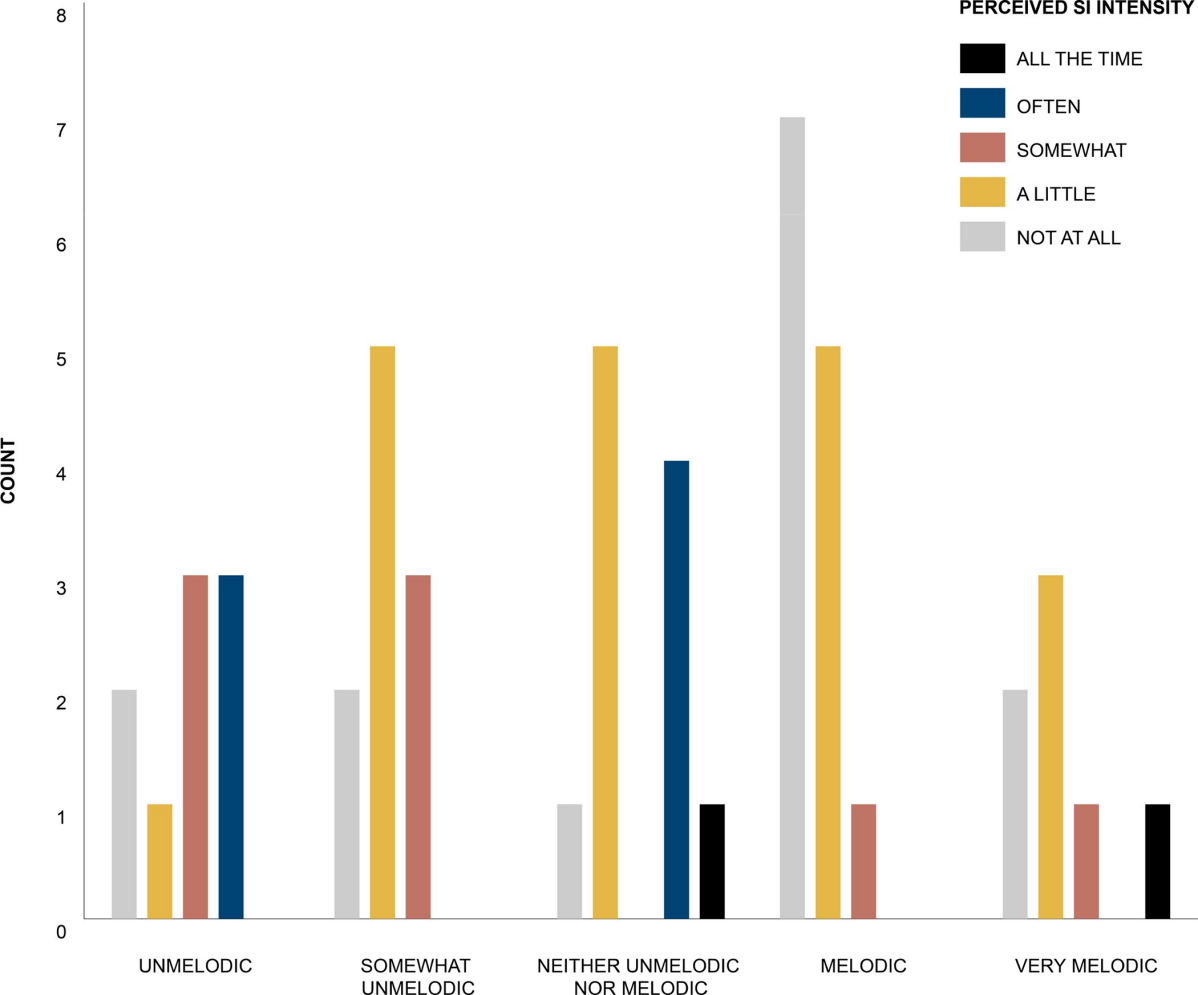
Counts of perceived SI intensity against participants reported waking sound melodic ranks.

Adjusted residuals of each cell revealed that those participants who returned a measure of perceived *SI* intensity as ‘Not at all’ and the waking sound melodicity as ‘Melodic’ were significantly more than expected under the null hypothesis of independence (*z* = 2.4, *p* = 0.022). Further, the measure of perceived *SI* intensity as ‘Often’ and the waking sound melodicity as ‘Neither unmelodic nor melodic’ were also observed to be significantly more frequent than expected under the null hypothesis (*z* = 2.4, *p* = 0.022). See Fig 3 for the counts of perceived *SI* intensity against participants reported waking sound melodic ranks.

### Secondary analysis

#### Test analysis No 8: Music element (Melody) vs Sound type

The primary contingency table analysis (Pearson’s Chi Square Test) showed that there was a significant relationship between the reported waking sound melodicity, and the subjects’ reported waking sound type *X*^*2*^ (12, *N* = 50) = 24.38, *p* = 0.018. This result was confirmed by the Fisher’s Exact Test *X*^*2*^ (*N* = 50) = 20.06, *p* = 0.013 with V = 0.37 corresponding to a moderately strong effect size. The adjusted residuals of each cell revealed several significant relationships between the reported melodicity of the subjects waking sound and the descriptions of the sound type under the null hypothesis of independence. See Table 7.

**Table 7:** Secondary analysis for adjusted residuals of music element—Melody vs sound type.

Test analysis no 8. results	*z* score	*p*-value
**Unmelodic vs Alarm Tone**	*z* = 2.9	*p* = 0.005
**Melodic vs Natural Sounds**	*z* = 2.4	*p* = 0.022
**Very Melodic vs Musical Song**	*z* = 2.1	*p* = 0.044

#### Test analysis No 9: Music element (Melody) vs Music element (Rhythm)

The contingency table analysis (Pearson’s Chi Square Test) showed that there was a significant relationship between the reported melody of the waking sound, and the reported waking sound rhythm *X*^*2*^ (16, *N* = 50) = 50.32, *p* < 0.001. This result was confirmed by the Fisher’s Exact Test *X*^*2*^ (*N* = 50) = 37.38, *p* < 0.001 with V = 0.50 corresponding to a strong effect size. The adjusted residuals of each cell revealed multiple significant relationship between the reported melody of the waking sound and rhythm of the waking sound under the null hypothesis of independence. These results are shown in Table 8.

**Table 8:** Secondary analysis for adjusted residuals of melody vs rhythm.

Test analysis no 9. results	*z* score	*p*-value
**Unmelodic vs Nonrhythmic**	***z* = 2.9**	***p* = 0.006**
**Somewhat unmelodic vs Somewhat nonrhythmic**	***z* = 2.3**	***p* = 0.028**
**Neither unmelodic nor melodic vs Neither nonrhythmic nor rhythmic**	***z* = 3.6**	***p* < 0.001**
**Melodic vs Rhythmic**	***z* = 3.7**	***p* < 0.001**
**Unmelodic vs Very rhythmic**	***z* = 2.9**	***p* = 0.006**
**Very melodic vs Very rhythmic**	***z* = 2.4**	***p* = 0.022**

## Discussion

This research is the first experimental online questionnaire to analyse waking audio with respect to *SI*. The study presents new insights to assist in the development, understanding, and future testing of waking sound for *SI* reduction, and extends existing research in the field of auditory countermeasures for *SI*. Considering the increasing demands in this 24-hour society, maximising human alertness by reducing *SI* can assist in this evolution, providing a safer environment in situations where performance is critical.

Existing research supports audio’s potential to counteract the intensity of *SI* [12, 33]. In this research context ‘excitative popular music’ has shown potential to negate *SI* [33], and provides the first insight into a musical sound types effect on *SI*, though many questions concerning the specific auditory mechanics and aesthetics required for the design and production of such stimuli remain unresolved. The present study investigates this subject through a self-report questionnaire, where an ecologically valid approach allows participants to submit responses from their preferred environment with minimal intervention. Through the questionnaire design and data obtained, we analyzed three primary research questions: (i) *‘Do waking sound types counteract the effects of SI*?*’*, (ii) *‘Do subjective feelings towards waking sound types counteract the effects of SI*?*’*, and (iii) *‘Do the musical elements of waking sound counteract the effects of SI*?’. In response to these primary research questions we discuss the key findings and secondary analysis results.

From the analysis specific to the key research questions: (i) ‘Do waking sound types counteract the effects of *SI*?’, and (ii) ‘Do subjective feelings towards waking sound types counteract the effects of *SI*?’, our results indicate that there is no significant relationship between the measure of perceived *SI* intensity and the participants waking sound type, or the subjects subjective feelings towards their waking sound type. With respect to key research question (iii), ‘Do the musical elements of waking audio counteract the effects of *SI*?’, our results revealed that those participants who reported a perceived intensity of *SI* as ‘Not at all’ and rated the waking sound melodicity as ‘Melodic’ where significantly more than expected under the null hypothesis of independence. Additionally, the perceived intensity of *SI* ranked as ‘Often’ and the waking sound melodicity as ‘Neither unmelodic nor melodic’ were also observed to be significantly more frequent than expected under the null hypothesis. These results indicate that waking audio reported as ‘Melodic’, irrespective of sound type, may possibly be associated to perceived *SI* reduction when compared to alternate melodicity rankings (Unmelodic, Somewhat unmelodic, Neither unmelodic nor melodic, and Very melodic). In contrast, waking stimuli interpreted as ‘Neither unmelodic nor melodic’, may perhaps act as a mechanism which intensifies perceived *SI* in comparison to descriptive counterparts (Unmelodic, Somewhat unmelodic, Melodic, and Very melodic). Therefore, in response to primary research question (iii), ‘Do the musical elements of waking sound counteract the effects of *SI*?’, we hypothesize that musical elements may be related to the perceived intensity of *SI*, however further research is required to interrogate these preliminary findings.

To rationalize this phenomenon is challenging considering both the lack of specific research in this context, and the absence of descriptive detail (including music elements) concerning auditory stimuli for waking. Research shows that sound can increase and maintain arousal, and attract human attention [5–11], however, music elements (specifically melody) in the context of counteracting *SI* is unknown. We hypothesize that stimuli perceived as melodically neutral may be interpreted as an auditory ambient variation of their counterpart (melodic). When compared to strong melodic material this neutral classification may be less likely to gain the human center of attention, may induce less arousal, and lead to reduced cognition, all of which are symptoms of *SI* [2, 3]. For situations where a stimuli’s melodic content is comparatively increased, we theorize that its auditory ambience transitions to salience, increasing arousal, cognition and attention, which may potentially lead to reduce effects of *SI*. For this analysis and building on available research, we propose that the melodic content of waking audio may be a musical factor that requires further research to determine its impacts on the effects of *SI*. Further, this result supports the requirement for detailed descriptions and inquiry of auditory test stimuli to inform analysis and discussion in this research field.

Given our finding that melodic content of waking audio may be a factor which influences perceived symptoms of *SI*, we now discuss this musical element and its relationship to waking sound types (e.g. Alarm tone and Natural sounds).

Our secondary analysis results show that an ‘Unmelodic’ waking sound has a significant relationship to the category ‘Alarm tone’ under the null hypothesis of independence; as does a ‘Very melodic’ report against ‘Musical song’; and ‘Melodic’ with respect to ‘Natural sounds’. From these results, we hypothesize that the musical mechanics and aesthetics of each sound type attributes to its perceived melodicity ranking. For example, as a function of the traditional sounding alarm (to wake sleeping humans through auditory intervention), by design this category of stimuli typically consists of a static rhythm, an insistent tonal center, and a salient aesthetic (e.g. a relentless beeping sound) [7, 8]. ‘Musical song’ incorporates melody as a dominant feature which is evident in popular Western music, examples include, yet are not limited to, The Beach Boys ‘Good Vibrations’ [102], and The Cures ‘Close to me’ [103]. The relationship between ‘Melodic’ and ‘Natural sounds’ is more difficult to define. One hypothesis we present is bird song at dawn (a ubiquitous ecological waking sound common in many cultures [7]) for their melodic qualities associated to a variety of species [104], and by virtue that birds typically exist in natural environments. Similarly to the investigation of music elements and sound types in this study, bird song is categorized through song ‘type’ and the ‘elements’ that articulate the song type. [105].

The quantity of rhythmic content in waking sound stimulus has a consistent and significant relationship to the perception of the reported melodicity (positive and negative) of waking audio under the null hypothesis of independence. Our results show that when the rhythmic content of the waking stimuli is perceived to decrease, the reported melodicity is reduced in turn. In contrast, as the rhythmic content of the waking sound is increased, the melodicity of the stimuli is perceived in this manner also. Additionally, the data reports an anomaly between the interpretation of ‘Very rhythmic’ and melodicity. Specifically, if the rhythmic content of the waking sound is described as ‘Very rhythmic’, then the perceived melodicity of the stimuli is reported as ‘Unmelodic’ and ‘Very melodic’ with equal significance. We hypothesize that this result is a by-product of the composition’s increased rhythmic elements, whereby a perceptual threshold is introduced, rendering the melodic and rhythmic interactions of the composition musically too complex for humans to clearly define as ‘Very melodic’ or ‘Unmelodic’. These results align with Boltz’s [106] findings suggesting an intrinsic relationship between melodic and rhythmic perception whereby temporal microstructures of compositions hold a primary role in the cognitive processing of melodic relations.

The demographic data collected from the respondents in this study produced two key findings for referencing in waking sound development. Firstly, a mobile phone is the most frequently reported device for communicating the participant’s waking sound (84%). Coupling this with estimates reporting that the audible frequency range of these devices ranges between 900 Hz– 10,000 Hz [107], this is central when developing auditory stimuli, as the human auditory hearing range spans 20Hz– 20,000 Hz [18]. Secondly, when considering the design of waking stimuli for *SI* and the aesthetic treatment of volume, 66% of subjects’ report that they employ a constant volume for waking. Specific research in this domain would clarify the benefits of auditory design targeting the most appropriate aesthetic volume treatment for waking sounds in the context of *SI*.

Our results indicate that musical elements may be related to the reduction of perceived *SI* intensity, however, further research is required in context to reinforce these understandings. Future investigations would benefit from a detailed investigation into the specific stimulus the participants utilize for waking, coupled with a reduced participation time post awakening (e.g. completing the test immediately after waking). Increasing subjective measures to identify in greater detail the participant sleep-wake attributes would assist in clarifying the presence and intensity of *SI* also. For example, Likert scales for hours slept, time of sleep, and the Karolinska Sleepiness Scale [108]. Further, a test battery could include objective measures (e.g. Psychomotor Vigilance Test [109]) to substantiate the subjective results obtained and allow comparisons. Primarily a technical limitation in the context of online testing, this obstacle is being minimized with the increasing availability of accessible software to accomplish this task. Gorilla [23] is one online software system enabling the design and production of online questionnaires with embedded interactive capabilities and data logging. Further, although 84% of our participants reported a moderate to high ability in describing music, we suggest that to clearly identify the musical aptitude of each participant, the research would benefit by incorporating a music test prior to undertaking the questionnaire. Additionally, considering the relatively unexplored nature of this study area, we did not seek to examine gender or age with respect to *SI* and awakening sound at this initial stage, however, future research may wish to consider these factors.

The primary analysis of this investigation showed a significant relationship between the melodicity of participants waking sound and a measure of perceived *SI*. By dissecting the musical elements of the waking sounds melodic content in the secondary analysis, we show that the rhythmic attributes of the audio have a significant relationship to the perceived melodicity of the stimulus. We define these results as key components for future examination to refine the understanding of how music and its elements in combination can be composed to produce the most effective waking sound stimuli to counteract *SI*.

## Supporting information

S1 Table Waking sound and sleep inertia questionnaire. (PDF)
