# Supplementary material for: Alarm tones, music and their elements: Analysis of reported waking sounds to counteract sleep inertia
Source: PLoS One. 2020 Jan 28;15(1):e0215788. doi: 10.1371/journal.pone.0215788 (PMC6986749; doi:10.1371/journal.pone.0215788)
Supplement: S1 Table — (PDF) [file pone.0215788.s001.pdf]

Alarm tones, music and their elements: Analysis of reported waking sounds to counteract sleep inertia.

Supporting information

'Waking Sound and Sleep Inertia' Questionnaire

Stuart J. McFarlane[1], Jair E. Garcia[1], Darrin S. Verhagen[2], Adrian G. Dyer[1*]

[1] School of Media and Communication, RMIT University, Melbourne, Vic, 3001, Australia.

[2] School of Design, RMIT University, Melbourne, Vic, 3001, Australia.

*adrian.dyer@rmit.edu.au (AGD)

Section 1: Welcome Page

Welcome to the 'Waking Sound and Sleep Inertia' test!

Please complete this questionnaire with in four hours after waking up.

By completing the questionnaire, you are acknowledging that you have given consent to participate in this questionnaire.

Thank you for your time.

Section 2: Demographic Information

Item 1. What is your age?

18 – 29	30 - 39	40 - 49	50 - 59	60+

Item 2. What is your gender?

Male	Female	Other

Item 3. How much music would you listen to on average per day?

0 - 30 minutes	31 minutes - 1 hour	1 - 3 hours	3 + hours

Item 4. Do you like listening to music?

Dislike a great deal	Dislike somewhat	Neither like nor dislike	Like somewhat	Like a great deal

Item 5. How well do you think you can describe music?

Not well at all	Slightly well	Moderately well	Very well	Extremely well

Section 3: Waking Sound

Item 6. Do you use audio to wake up?

Yes - Forward to Item 7.	No - Forward to Item 6.1.

Item 6.1. How do you wake up?

Please describe:
Forward to Item 14.

Item 7. What device do you use to play the audio?

Clock Radio (alarm)	Mobile phone	Tablet	Laptop	Other - Please describe:

Item 8. How many days a week do you use audio to wake up?

1.	2.	3.	4.	5.	6.	7.

Item 9. Do you use the same audio to wake up every day?

Yes	No – Please describe:

Item 10. Which most frequently used audio for waking up best represents yours?

Alarm tone	Musical song<br>Forward to 10.1.	Instrumental music<br>Forward to 10.1.	Natural sounds	Radio<br>Please specify the radio station:	Other<br>Please describe:

Item 10.1. Please specify the genre:

Rock	Pop	Hip Hop/ Rap	Disco	Electronic	Country	Blues	Classical	Opera	R&B / Soul	World music

Item 11. On a scale from 1 to 5; how would you classify the audio's...

	1.	2.	3.	4.	5.
Melody (unmelodic = 1, neither unmelodic nor melodic = 3, very melodic = 5)					
Rhythm (non-rhythmic = 1, neither nonrhythmic nor rhythmic = 3, very rhythmic = 5)					
Tempo (slow tempo = 1, neither slow tempo nor fast tempo = 3, fast tempo = 5)					
Pitch (low pitch = 1, neither low pitch nor high pitch = 3, high pitch = 5)					
Volume (low volume = 1, neither low volume nor high volume = 3, high volume= 5)					

Item 12. Does of the volume increase in loudness over time?

Yes	No

Item 13. How would you classify the feeling of the audio when waking up?

Very unpleasant	Unpleasant	Neutral	Pleasant	Very pleasant

Section 4: Sleep Inertia

Item 14. SIQ. After you wake up, to what extent do you…

	1 = Not at all	2 = A little	3 = Somewhat	4 = Often	5 = All the time
Have problems getting out of bed?					
Hit the snooze button on the alarm?					
Bump into and drop things?					
Notice that you move more slowly?					
Notice that you feel sleepy?					
Notice your eyes feeling heavy, sore, or itchy?					
Notice your arms and/or legs feeling tired or heavy?					
Notice that your mind feels groggy, fuzzy or hazy?					
Notice that it is difficult to keep your balance?					
Have difficulty concentrating?					
Find that you think more slowly?					
Find that you make more mistakes/errors?					
Have difficulty getting your thoughts together?					
